# Mini-review on laser-induced nanoparticle heating and melting

**DOI:** 10.3389/fchem.2024.1463612

**Published:** 2024-11-06

**Authors:** Ilya V. Baimler, Alexander V. Simakin, Alexey S. Dorokhov, Sergey V. Gudkov

**Affiliations:** ^1^ Prokhorov General Physics Institute of the Russian Academy of Sciences, Moscow, Russia; ^2^ Federal State Budgetary Scientific Institution “Federal Scientific Agroengineering Center VIM” 5 (FSAC VIM), Moscow, Russia; ^3^ Institute of Biology and Biomedicine, Lobachevsky State University of Nizhny Novgorod, Moscow, Russia

**Keywords:** nanoparticles, laser radiation, laser fragmentation, laser heating, laser melting

## Abstract

The development of various nanomaterials production technologies has led to the possibility of producing nanoparticles (NPs) and nanostructures, which can find a wide range of applications, from the fabrication of microelectronic devices to the improvement of material properties and the treatment of cancer. The unique characteristics of nanoparticles are primarily due to their small size, which makes size control important in their preparation. Modification of nanoparticles by laser irradiation and obtaining desired nanoparticle properties is a promising approach because of its ease of implementation. The purpose of this review is to analyze the works devoted to the study of laser-induced heating and melting of nanoparticles, to collect information and evaluate the results of using this method for functionalization and modification of metallic nanoparticles, and to discuss promising directions for the use of this technique.

## 1 Introduction

Nowadays, nanoparticles play a key role in science and are widely used in various fields of industry (Alsaba et al., 2020), agro-technology (Kaningini et al., 2022; Gudkov et al., 2024), biochemistry (Rico et al., 2015), biophysics (Moore and Chow, 2021) and catalysis (Tack et al., 2024). Synthesis and modification of nanoparticles by laser irradiation is a well-known method for fabrication of nanoparticles with high purity surface in liquids using laser pulses, which has proven to be an environmentally friendly, simple and convenient method for obtaining nanoscale objects (AlMalki et al., 2022; Jiang et al., 2022; Bhardwaj et al., 2020).

Due to their unique optical properties, nanoparticles are well suited for heating by laser radiation (Qin and Bischof, 2012). In this context, colloidal particles are of particular interest, since conventional heating methods limit the maximum achievable temperature to the boiling point of the solvent. Colloidal particle solutions modified by laser heating and melting can potentially be used in various fields of nanotechnology, ranging from biological imaging (Saha et al., 2012), drug delivery (Wilczewska et al., 2012), water purification (Kefeni et al., 2017) to the generation of high-frequency mechanical vibrations (Pelton et al., 2009).

The heating of metallic nanoparticles is based on the absorption of light by the nanoparticles and it occurs in the following way: free electrons absorb photon energy within a time scale of 100 femtoseconds (Heilweil and Hochstrasser, 1985; Ekici et al., 2008). After that, the equilibrium state is reached through electron-electron relaxation within 10–100 femtoseconds (Heilweil and Hochstrasser, 1985; Hodak et al., 1998). Due to the electron-phonon interaction that occurs between 100 femtoseconds and picoseconds, the temperature of the particle increases (Letfullin et al., 2008). Finally, the increase in temperature in the surrounding environment occurs due to energy transfer between the particles and the surrounding medium through phonon-phonon interactions that take place over a range from picoseconds to nanoseconds (Ekici et al., 2008). It has been shown that certain parameters, such as laser fluence (Pustovalov et al., 2008; Nedyalkov et al., 2011), pulse duration (Pustovalov, 2006), wavelength (Astafyeva et al., 2017), repetition rates (Sobhan et al., 2010), as well as nanoparticle size (Duan et al., 2018) and material (Pyatenko et al., 2013) affect the threshold values for the onset of melting of nanoparticles and their heating rates.

Melting is a well-known and well-studied phenomenon that continues to reveal new aspects when it occurs under laser irradiation of nano-objects (Nanda, 2009). Recent experimental data on the anomalously slow nanosecond melting process of thin gold films under the influence of femtosecond laser pulses have prompted studies aimed at understanding the mechanisms underlying this phenomenon (Arefev et al., 2022).

The key parameter when considering the processes of particle heating under laser irradiation is the amount of heat loss from nanoparticles to the surrounding solvent due to conduction, convection, and radiation heat transfer (Wang et al., 2012). It has recently been shown that heat transfer to the surrounding particle environment is not solely determined by the thermal conductivity of the surrounding fluid. Particle cooling occurs by heat transfer through the vapor/liquid interface (Plech et al., 2004). In this case, the characteristic length of thermodiffusion can play a crucial role in the formation of nanoparticles and bulk materials using laser pulses (Llamosa et al., 2013).

When a nanoparticle is exposed to excessively intense laser irradiation, the energy of which exceeds the melting and ionization energies, it passes into the state of nanoplasma, which is located in a shell of liquid vaporized during heating, which is accompanied by a number of nonequilibrium dynamical processes (Niozu et al., 2021). It is worth noting that ionization mechanisms, as studies show, depend on the pulse duration - multiphoton photoionization is characteristic of femtosecond pulses, and avalanche ionization is characteristic of pico- and nanosecond pulses (Noack and Vogel, 1999; Linz et al., 2016; Yang et al., 2023). The formation of a thermally induced vapor-gas shell around the particle plays one of the key roles in the processes of changing the morphology and size of the particle (Metwally et al., 2015; Lombard et al., 2014).

At present, the mechanisms of heat transfer between nanoparticles and liquids in the context of nanoparticle wetting phenomena are poorly understood (Tascini et al., 2017). Unwanted thermal effects are another problem (Hashemi et al., 2019). In order to prevent unwanted heating, it is necessary to investigate the localized heating effect of particles. The peculiarity of the study of this problem lies in the difficulty of determining temperatures, since the accurate determination of temperature in the nanoscale region has a number of peculiarities (Liu and Liu, 2019).

A promising area of research in nanomedicine involves the use of nanoparticles that can accumulate in disease foci for drug delivery or thermal destruction of cells. Nanoparticles entering diseased tissues are activated by laser radiation, which initiates heat transfer by the particle and changes the temperatures of the medium (Riley and Day, 2017; Day et al., 2009). It is assumed that heat can be controlled and focused at the nanoscale, which allows precise control of the processes that occur (Terentyuk et al., 2009; Sokolovskaya et al., 2021).

**FIGURE 1:**
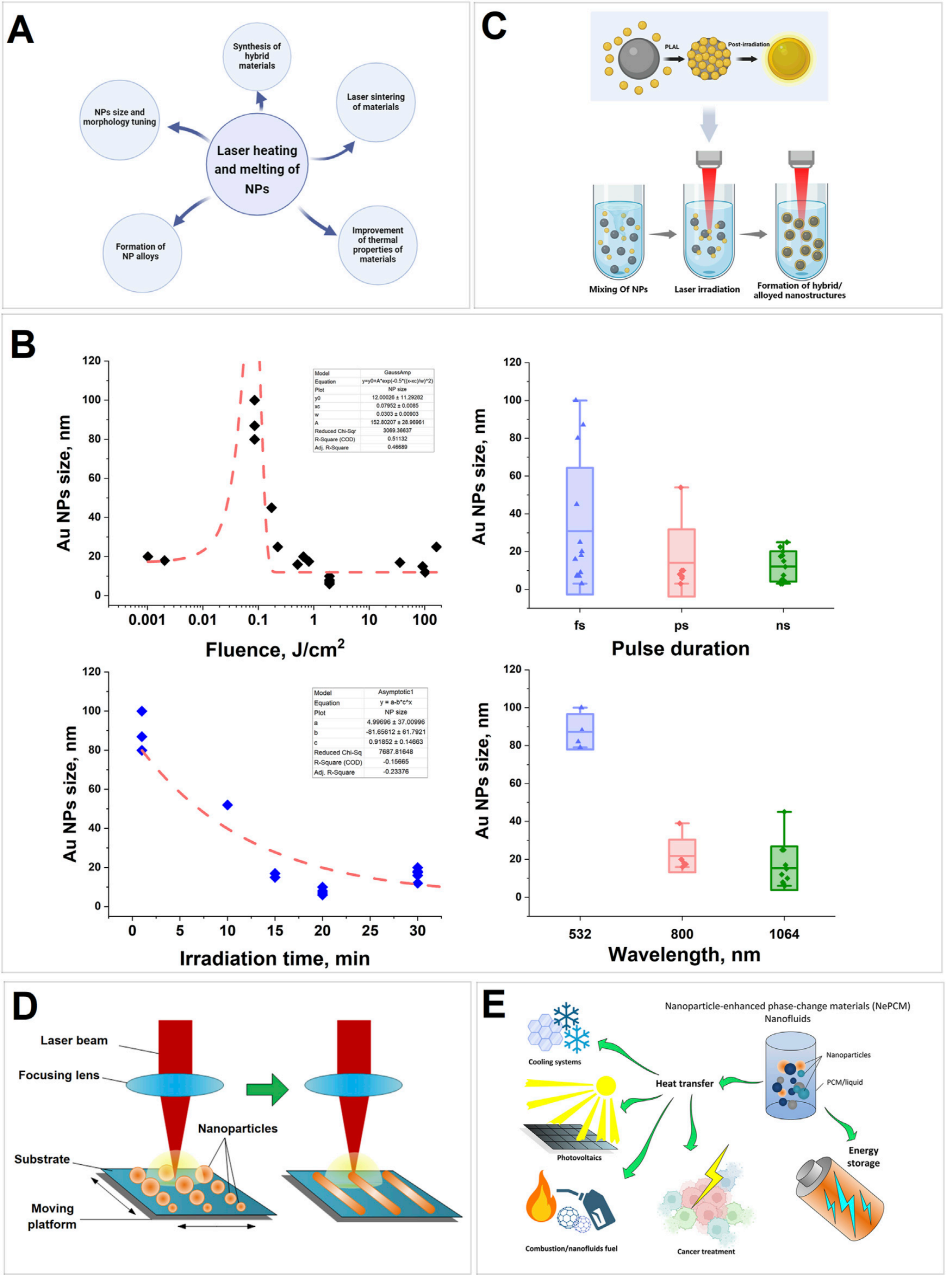
**(A)** Schematic representation of the application of laser heating of nanoparticles; **(B)** Dependence of size of Au nanoparticles upon laser-induced irradiation of colloids during laser fragmentation in aqueous solutions depending on laser fluence, pulse duration, irradiation time and wavelength; **(C)** A schematic representation of the synthesis process for nanocomposite materials through laser irradiation of nanoparticle colloids in a liquid medium; **(D)** Scheme of the basic process using laser sintering of nanoparticles; **(E)** Diagram of possible applications for the nanoparticle-enhanced materials.

The processes of particle heating by laser radiation are also involved in such technologies as selective laser melting (SLM) (Liu et al., 2011), laser sintering, creation of hybrid materials and alloys (Xiao et al., 2020). Laser-induced heating is widely used to modify and form nanostructures on metal surfaces (Kucherik et al., 2021; Zhu et al., 2016; Yadavali et al., 2016; Sdvizhenskii and Lednev, 2022). All the techniques related to the heating and melting of nanoparticles under the influence of laser radiation, which will be discussed in this review, are presented in Figure 1A.

One of the most popular nanoparticle materials used in nanotechnology is gold. Gold nanoparticles possessing surface plasmon resonance are currently used as promising heating centers in various chemical and medical applications (Qin and Bischof, 2012; Gorin et al., 2008; Yang et al., 2019). However, the suitable wavelength for heating nanoparticles, i.e., the surface plasmon resonance wavelength, is only in the visible spectrum, and the maximum achievable temperature is limited by their relatively low melting point. The use of other materials as potential heating centers for nanoparticles will circumvent these problems.

This review discusses the current state of knowledge on the technology of laser-induced melting of metal nanoparticles. The advantages and potential problems of this technology, current applications, and promising directions for further development are discussed.

## 2 Control of the sizes and morphology of nanoparticles during the heating process

Controlling the shape of nanoparticles is an important aspect to adjust the parameters of nanoparticle technologies. For example, the shape of the particles can influence the signal of nanoparticle-enhanced laser breakdown spectroscopy (NELIBS). Experimental results show that silver nanowires and nanocubes enhance LIBS signals compared to spherical particles (Abdelhamid et al., 2020). The irradiation of colloids by laser irradiation can reduce the size of nanoparticles and decrease the width of the size distribution. The final particle size and distribution width can be controlled by varying the irradiation intensity (Mafuné et al., 2001). The application of laser pulse fragmentation in liquid (LFL) method can produce stable colloidal gold nanoparticles in water (Ashikkalieva et al., 2022). The final size of fragmented nanoparticles is mainly determined by the exposure time and radiation intensity (Vasa et al., 2014). Coating plasmonic nanoparticles with temperature-sensitive polymers allows tracking the temperature increase during laser heating by monitoring the shift of the plasmon resonance peak and absorption spectrum, which are affected by the temperature-dependent refractive index of the medium (Honda et al., 2011; Mikami et al., 2021).

The technology of laser-induced heating also makes it possible to change the morphology of nanoparticles. It was found that irradiation of ellipsoidal gold nanoparticles results in their transition to spherical shape at temperatures much lower than their melting temperature. The effect of particle shape change may be due to partial surface melting (Inasawa et al., 2005). A method of producing spherical porous gold nanoparticles on glass substrates using ultraviolet laser followed by chemical selective etching is reported. This method allows the creation of branched nanometer-scale structures inside spherical particles (Schmidl et al., 2022).

The choice of the required laser intensity and wavelength allows selectively obtaining particles of the desired shape - spheres, nanorods, nanoprisms, one-dimensional structures, nanochains, etc. By adjusting the wavelength of radiation, it is possible to choose the spatial extent of the ensembles of heated nanoparticles, which allows controlling the morphology of nanostructures formed during melting (Tarasenko et al., 2005; Catone et al., 2018; Ashikkalieva et al., 2023). Laser size reduction and formation of nanostructures from gold nanoparticles is a promising method for producing nanoscale devices containing nanoparticles and nanowires of desired sizes (Mafuné et al., 2003; Simakin et al., 2021; Kirichenko et al., 2012; Serkov et al., 2015a; Serkov et al., 2015b).

An important factor in particle heating is the duration of the radiation pulse. The use of nanosecond pulses leads to either fragmentation or complete melting of the material and the formation of spherical-shaped particles, while energetically the melting threshold was higher by two orders of magnitude compared to femtosecond pulses (Link et al., 2000a). Irradiation of gold nanoparticles with picosecond pulses of gold nanoparticles at a wavelength of 532 nm shows lower heating efficiency compared to other wavelengths (Plech et al., 2022). It is suggested that the decrease in heating efficiency can be attributed to the effects of scattering enhancement, thermoelectronic emission, plasma formation, plasmon interaction with the surroundings and the effects of partial lattice melting and deformation. It was found that femtosecond laser pulses are more suitable for the photothermal formation of gold nanorods, since the energy transfer to the crystal lattice occurs faster than the characteristic electron-phonon relaxation time (Link et al., 2000a).

Plasmonic nanoparticles can be effectively heated when exposed to femtosecond laser pulses with very low energy densities (Huang et al., 2015). It is shown that the efficiency of particle heating under these conditions depends on the local geometry of each nanoparticle and the polarization of the incident laser radiation.

In some cases, laser-induced heating of particles allows the material of nanoparticles to be transferred to other phase states. For example, when colloidal dispersions of copper oxide nanoparticles were irradiated with nanosecond radiation with a wavelength of 532 nm, phase transitions from copper (II) oxide to crystalline copper were observed in nanoparticles. The phase transition was limited to the minimum particle size (23–29 nm), due to more efficient heating process, less cooling effect (Kranz et al., 2022). X-ray diffraction study of MnO nanoparticle samples showed that laser heating led to changes in the existing phases of the sample, including the destruction of the MnO phase and the formation of new phases such as MnO_2_, Mn_3_O_4_, and MnOOH, as well as the deposition of Mn^2+^ ions on the sample surface (Hadžić et al., 2018). Laser heating and fragmentation of selenium nanoparticles leads to the formation of crystalline selenium in new phases (Varlamova et al., 2023; Baimler et al., 2024; Singh et al., 2010; Poborchii et al., 1999; Sakaguchi and Tamura, 2021).

The potential of submicron spherical boron carbide-based B_4_C boron carbide particles as nanoscale heating agents is currently being investigated because B_4_C has a broader optical absorption spectrum and a higher melting point than gold (Pavlov et al., 2022; Aiyyzhy et al., 2022a; Mayelifartash et al., 2021; Stone et al., 2024). The experiment demonstrated that B_4_C particles exhibit a significant response in the wavelength range of 300–1,100 nm and are capable of acting as a nanoscale heater at temperatures exceeding 2000 K, which can be utilized in the design of volume-selective heating agents (Kojima et al., 2020).

During heating and melting of nanoparticles, the formation of larger particles formed by the fusion of several molten particles is sometimes observed. A key role in the formation of large submicron particles is played by the vapor-gas shell surrounding the particles during heating and boiling of the surrounding liquid (Tabayashi et al., 2021). It was found that the use of high pressures in laser irradiation of colloids leads to a decrease in the size of nanoparticles, which is associated with the processes of gas bubble formation and changes in the dynamics of heat loss in heated particles (Werner and Hashimoto, 2013; Wei and Saitow, 2012). Wettability affects the thermal conductivity of the nanoparticle-water interface. Greater wettability enhances the interaction of low-frequency phonon modes at the solid-liquid interface, thereby increasing the efficiency of thermal energy transfer, as has been shown for Fe particles (Ma et al., 2024a). The thermal effect was found to be the main reason for the transformation of the atomic structure of single-crystal gold nanoparticles. Nanoparticles with defects in the crystal structure show increased surface activity due to low coordination number (Zhu et al., 2021).

The process of laser heating of nanoparticles is studied using analytical methods and modeling. A significant part of the recently published review (Pustovalov, 2024) is devoted to modeling of the processes of heating by laser radiation of individual particles and their aggregates.

The study of diffraction profiles of nanoparticles and their atomistic modeling during their laser fragmentation by picosecond laser radiation allowed to identify the main stages of the process of nanoparticle heating and fragmentation. At low laser intensity, a short-term overheating of crystalline nanoparticles above the melting temperature, melting, subsequent cooling and solidification are observed. When the radiation energy density is three times the melting threshold, fragmentation begins with the evaporation of gold atoms and their subsequent condensation into small nanoparticles. When the energy density increases to more than five times the melting threshold, there is a transition to rapid (explosive) phase destruction of superheated nanoparticles into small liquid droplets and vaporized atoms (Plech et al., 2023). Atomistic modeling also shows that the combination of lattice superheating and laser-induced stress relaxation ensures the predominance of the homogeneous melting process at all energy levels below the melting threshold, keeping the melting duration at approximately 100 picoseconds or less (Arefev et al., 2022).

The cluster-based molecular dynamics of the two-temperature model becomes an effective method to study the microscopic dynamics of nanoparticles. The two-temperature model, integrated with the molecular dynamics model and the localized melting model, effectively simulates the energy transfer and relaxation processes that underlie the final size and morphology of nanoparticles (Chen et al., 2014; Cui et al., 2013; Alavi and Thompson, 2006; Shibuta and Suzuki, 2007).

The results of laser-induced irradiation of nanoparticle colloids in water are given in Table 1.

**TABLE 1:** List of experimental works on synthesis, structuring and modification of nanoparticles using laser-induced irradiation.

NPs material	Experimental setup	Wavelength	Pulse duration	Fluence	Frequency	Irradiation time	NP size	NP form	References
		nm	s	J/cm^2^	Hz	min	nm		
Se	LAL	510	1 × 10^−8^	65.07861	15,000	100	60	Spherical	Kuzmin et al. (2012)
B	LAL	1,060	2 × 10^−7^	141.4752	20,000	200	32	Spherical	Aiyyzhy et al. (2022b)
TiO_2_	LAL	532	1 × 10^−11^	0.565,901	50,000	60	80	Spherical	Serkov et al. (2015c)
TiO_2_	LAL	532	1 × 10^−8^	0.565,901	50,000	60	20	Elongated	
Si	LAL	510	2 × 10^−8^	1.273,277	15,000	30	84	Spherical	Dolgaev et al. (2002)
Si	LAL	510	2 × 10^−8^	1.273,277	15,000	30	80	Spherical	
Si	LAL	510	2 × 10^−8^	1.273,277	15,000	30	74	Spherical	
Si	LAL	510	2 × 10^−8^	0.763,966	15,000	30	74	Spherical	
Si	LAL	510	2 × 10^−8^	0.763,966	15,000	30	60	Spherical	
TiO_2_	LAL	510	2 × 10^−8^	4.074487	15,000	30	35	Spherical	
Ag	LAL	510	2 × 10^−8^	1.273,277	15,000	30	60	Nanodiscs	
Ag	LAL	1,030	2 × 10^−8^	1.909,916	50,000	20	26	Spherical	Saraeva et al. (2019)
Ag	LAL	1,030	4.1 × 10^−12^	1.909,916	50,000	20	26	Spherical	
Ag	LAL	1,030	8.6 × 10^−12^	1.909,916	50,000	20	25	Spherical	
Si	LAL	1,030	3 × 10^−13^	1.909,916	50,000	20	51	Spherical	
Si	LAL	1,030	4.1 × 10^−12^	1.909,916	50,000	20	45	Spherical	
Si	LAL	1,030	8.6 × 10^−12^	1.909,916	50,000	20	45	Spherical	
Ag	LAL	1,064	1 × 10^−11^	1.325,778	50,000	-	20	Spherical	Barcikowski et al. (2007)
Ag	LAL	800	1.2 × 10^−13^	1.325,778	5,000	-	45	Spherical	
Ag	LAL	800	1.2 × 10^−13^	2.784,134	5,000	-	35	Spherical	
Ni	LAL	800	1.2 × 10^−13^	90.54415	1,000	60	8	Spherical	Muñeton Arboleda et al. (2015)
C	LAL	532	6 × 10^−9^	12.73277	10	60	30	Spherical	De Giacomo et al. (2011)
C	LAL	532	6 × 10^−9^	12.73277	10	60	30	Elongated	
C	LAL	532	6 × 10^−9^	12.73277	10	60	25	Elongated	
ZnO	LAL	532	1 × 10^−8^	4.053339	10	5	19	Spherical	Guillén et al. (2015)
ZnO	LAL	532	1 × 10^−8^	4.053339	10	5	25	Spherical	
ZnO	LAL	532	1 × 10^−8^	4,053,339	10	5	28	Spherical	
ZnO	LAL	532	1 × 10^−8^	4.053339	10	5	100	Nanoflakes	
ZnO	LAL	532	1 × 10^−8^	5.976,607	10	5	100	Nanoflakes	
ZnO	LAL	532	1 × 10^−8^	8.705,521	10	5	33	Spherical	
ZnO	LAL	532	1 × 10^−8^	5.976,607	10	5	35	Spherical	
ZnO	LAL	532	1 × 10^−8^	8.705,521	10	5	25	Spherical	
ZnO	LAL	532	1 × 10^−8^	5.976,607	10	5	30	Spherical	
ZnO	LAL	532	1 × 10^−8^	8.705,521	10	5	29	Spherical	
ZnO	LAL	532	1 × 10^−8^	5.976,607	10	5	21	Spherical	
ZnO	LAL	532	1 × 10^−8^	8.705,521	10	5	21	Spherical	
Au	LAL	510	2 × 10^−8^	1.273,277	15,000	30	80	Elongated	Dolgaev et al. (2002)
Au	LAL	1,030	3 × 10^−13^	1.909,916	50,000	20	7	Spherical	Saraeva et al. (2019)
Au	LAL	1,030	4.1 × 10^−12^	1.909,916	50,000	20	8	Spherical	
Au	LAL	1,030	8.6 × 10^−12^	1.909,916	50,000	20	10	Spherical	
Au	LAL	1,030	3 × 10^−13^	1.909,916	50,000	20	7	Spherical	
Au	LAL	1,030	4.1 × 10^−12^	1.909,916	50,000	20	7	Spherical	
Au	LAL	1,030	8.6 × 10^−12^	1.909,916	50,000	20	6	Spherical	
Au	LAL	800	1.2 × 10^−13^	0.085735	5,000	1	80	Spherical	Menéndez-Manjón et al. (2010)
Au	LAL	800	1.2 × 10^−13^	0.085735	5,000	1	87	Spherical	
Au	LAL	800	1.2 × 10^−13^	0.085735	5,000	1	100	Spherical	
Au	LFL	1,064	9 × 10^−7^	91.67595	20,000	15	15	Spherical	Simakin et al. (2019)
Au	LAL	1,064	1 × 10^−8^	101.8622	10,000	30	12	Spherical	Simakin et al. (2021)
Au	LFL	1,064	1 × 10^−8^	162.6965	10,000	60	25	Elongated	
Au	LAL	532	1 × 10^−11^	1.273,277	50,000	30	10	Spherical	Barmina et al. (2014)
Au	LFL	1,025	4.5 × 10^−13^	0.018335	1,000	45	9	Spherical	Maximova et al. (2015)
Au	LFL	1,025	4.5 × 10^−13^	0.112,048	1,000	45	7	Spherical	
Au	LFL	1,025	4.5 × 10^−13^	0.173,166	1,000	45	45	Spherical	
Au	LFL	1,025	4.5 × 10^−13^	0.224,097	1,000	45	25	Spherical	
Au	LFL	515	2 × 10^−13^	0.050931	10,000	-	3	Spherical	Bongiovanni et al. (2021)
Au	LFL	532	9 × 10^−9^	1.65526	100	-	3	Spherical	Ziefuß et al. (2018)
Au	LFL	532	7 × 10^−9^	2.164,571	2000	-	3	Spherical	
Au	LFL	532	1 × 10^−11^	0.031832	80,000	-	3	Spherical	
Au	LAL	1,064	1 × 10^−11^	0.031832	100,000	10	54	Spherical	
Au	LAL	1,064	1 × 10^−8^	35.65176	10	15	18	Spherical	Tsuji et al. (2013)
Au	LFL	532	8 × 10^−9^	0.061117	10	30	234	Spherical	
Au	LFL	532	8 × 10^−9^	0.040745	10	30	181	Spherical	
Au	LFL	532	8 × 10^−9^	0.08149	10	30	255	Spherical	
Au	LFL	532	8 × 10^−9^	0.101,862	10	30	309	Spherical	
Au	LFL	800	1 × 10^−13^	10.15049	1,000	30	7.5	Spherical	Link et al. (2000b)
Au	LFL	800	1 × 10^−13^	0.509,311	1,000	30	16	Spherical	
Au	LFL	800	1 × 10^−13^	0.002037	1,000	30	18	Spherical	
Au	LFL	800	1 × 10^−13^	0.001019	1,000	30	20	Spherical	
Au	LFL	800	1 × 10^−13^	0.000204	1,000	30	40	Spherical	
Au	LFL	800	7 × 10^−9^	16.82226	1,000	30	7.5	Spherical	
Au	LFL	800	7 × 10^−9^	4.15764	1,000	30	4	Spherical	
Au	LFL	800	7 × 10^−9^	0.814,897	1,000	30	17.5	Spherical	
Au	LFL	800	7 × 10^−9^	0.649,631	1,000	30	20	Spherical	
Au	LAL	532	5 × 10^−9^	1.591,596	10	20	22.5	Spherical	Fazio et al. (2020)
Au	LFL	532	5 × 10^−9^	1.273,277	10	120	5	Spherical	
Au	LFL	800	4 × 10^−14^	-	1,000	30	5	Spherical	Okamoto et al. (2019)
Ag	LAL	800	1.2 × 10^−13^	1.38	1,000	-	50	Spherical	Barcikowski et al. (2007)
Ag	LAL	800	1.2 × 10^−13^	2.86	1,000	-	20	Spherical	
Ag	LAL	1,064	1 × 10^−11^	1.39	50,000	-	35	Spherical	
Au	LFL	800	1.3 × 10^−13^	-	100	130	15	Spherical	Akman et al. (2013)

Analysis of the results of experimental work on laser irradiation and laser fragmentation of gold colloids in water shows that the main parameters determining the particle size are fluence, pulse duration, pulse repetition rate and irradiation duration (Figure 1B). Changes in fluence can lead to changes in the shape of gold particles (0.001–0.01 J/cm^2^), their melting and aggregation into larger particles (0.1 J/cm^2^), and fragmentation and formation of smaller particles (1–100 J/cm^2^). The use of femtosecond pulses demonstrates the possibility of obtaining particles in a wider range of sizes compared to pico- and nanosecond pulses. Increasing the irradiation time and radiation frequency increases the number of interactions of laser pulses with particles, which leads to a decrease in size. Irradiation of colloids at a wavelength coinciding with the Au plasmon resonance wavelength leads to more efficient melting and formation of large aggregates.

## 3 The formation of nanostructured alloys and hybrid materials through laser melting of nanoparticles

Significant interest is emerging in the development of alloy metallic nanoparticles, due to their synergistic effect and because of their unique hybrid characteristics. Alloy nanoparticles are known to have higher catalytic activity than their monometallic counterparts (Xing et al., 2023; Khan et al., 2020; Jiang et al., 2023). Current research is focused on the creation of metal oxide nanostructures based on CuO, ZnO, TiO₂ and Fe₂O₃ (Pembere et al., 2022; Mintcheva et al., 2020; Nag et al., 2023; Omelchenko et al., 2015). Irradiation of a mixture of nanoparticle colloids of different materials allows the preparation of new nanocomposite materials (Golubovskaya et al., 2024; Fakhrutdinova et al., 2024). For example, irradiation of a mixture of colloids of two different nanoparticle plasmonic materials of gold and silver with femtosecond pulses allows to obtain Au-Ag nanocomposites, the formation of which involved the mechanism of laser melting and doping (Hidayah and Herbani, 2020). The formation of Au/MxOy (M = Fe, Co, Ni) composite nanoparticles with different morphology and sizes was observed by laser irradiation of particle colloids during their mixing (Swiatkowska-Warkocka et al., 2017). A schematic representation of the process of creating hybrid/alloy nanomaterials is shown in Figure 1C. It is possible to form alloy nanoparticles and hybrid materials by laser-induced irradiation of double thin films of metals using laser radiation. For example (Dzienny et al., 2022), describes a method for producing Au-Sn particles by laser-induced dewetting. In Kovalev et al. (2023) the technology of hyperdoping of silicon films with gold by irradiation of Au and Si double films with nano- and picosecond radiation is reported. In Hodges et al. (2017), a technique for synthesizing three-component Ag-Pt-Fe_3_O_4_ and Au-Pt-Fe_3_O_4_ heterotrimers is described. In Amendola et al. (2017) it is reported about obtaining Au-Fe alloy composite nanoparticles by laser ablation of multilayer gold and iron films of different thickness in ethanol and water. In some cases, the formation of alloy particles occurred at room temperature by simple mixing of colloids, as shown in Křenek et al. (2022), where TiSi_2_ nanoparticles were obtained.

## 4 Laser sintering of metal nanoparticles

Laser sintering has been a well-established method for several years and is widely used with continuous and pulsed lasers of various durations. This technology is widely used in the production of electronics devices. The characteristics of devices fabricated by this method depend to a large extent on the sintering conditions, melt state, laser radiation parameters, particle structure and substrate condition (Chen et al., 2023). A schematic representation of the basic laser sintering process is shown in Figure 1D. Using the low-temperature sintering method, stable Cu@Ag nanoparticles in the form of nanoribbons were synthesized, which may have practical applications in flexible printed electronics (Zhang et al., 2022). Laser sintering has been reported to produce ruby particles by irradiating Al_2_O_3_ and Cr_2_O_3_ powders in quasi-continuous mode (Aiyyzhy et al., 2023). The ruby particles obtained by the authors were then used in the manufacture of photoconversion coatings for greenhouses (Paskhin et al., 2023).

Laser-induced forward transfer (LIFT) and selective laser sintering (SLS) are two promising technologies based on the process of laser heating of materials that can be used to create a conductive layer of metallic nanoparticle ink on various substrates (Lim et al., 2020).

Selective laser melting (SLM) is a laser additive manufacturing technique based on the principle of layer-by-layer material deposition. SLM is used to fabricate various materials including alloys of different metals (Lu and Zhuo, 2023; Lu et al., 2023; Ma et al., 2024b; Sajjadi et al., 2024) and composite materials (Xi et al., 2021; Erutin et al., 2023).

Despite the extensive literature on conventional laser sintering methods, the use of ultra-short femtosecond pulses in this technology remains a relatively unexplored area (Sharif et al., 2022).

## 5 Nanoparticle enhanced thermal properties of materials

Incorporation of nanoparticles into various materials demonstrates significant changes in the thermal properties of the starting material. A new class of substances, nanofluids, have recently become the object of close attention due to a number of unique properties. Nanofluids are a mixture of nanoparticles and a solvent (Das et al., 2006). Nanofluids have been shown to significantly improve the thermal properties of basic solvents (Kumar et al., 2018). The improvement in the heat transfer properties of nanofluids has led to interest in their study and use in various engineering applications. These include nuclear technology (Buongiorno et al., 2008), desalination (Iqbal et al., 2021), machining (Ramesh and Prabhu, 2011), and cooling (Rafati et al., 2012). They are also used in solar energy (Izadi and Assad, 2024) and electron cooling (Moita et al., 2021).

Another promising class of materials, phase-change materials (PCM), can be used for heat storage and transfer (Chen et al., 2020). The addition of nanoparticles to these materials (NPCM) has been demonstrated to increase the thermal conductivity of the material (Khodadadi et al., 2013; Colla et al., 2017; Krishna et al., 2017) and change the phase transition temperatures (Lin and Al-Kayiem, 2016; Munyalo and Zhang, 2018). Laser heating and the addition of nanoparticles to PCMs will allow for a faster transition of the material into the crystalline phase (Kozyukhin et al., 2019). A diagram illustrating possible applications of the materials described is presented in Figure 1E.

In perspective, nanoparticles could be used to alter the properties of a wider range of materials. It has been shown that the volume of the melt region and the size of the heat affected zone during the melting and solidification processes of materials can be controlled by adding aluminum and silicon carbide nanoparticles to the material (Ma et al., 2017).

## 6 Conclusion

This mini-review provides a brief overview of recent advances in research and technology based on the heating and melting of nanoscale particles under the action of laser radiation. Nanoparticles themselves are of great interest for study and use in various fields ranging from medicine, agricultural engineering, catalysis to the creation of electronic devices. Moreover, with the help of laser radiation it is quite easy to change the key characteristics of nanoparticles (shape and size), to create new materials based on particles, to change the thermal characteristics of materials thereby expanding the possibilities of nanomaterials application. Overall, promising areas for research in this field will include the study of the interactions between nanoparticles and femtosecond laser pulses. Additionally, there is an interest in using these pulses to create novel hybrid materials such as nanocomposites and nanoparticles-based alloys through the use of the effect of localized surface melting of nanoparticles. Furthermore, the development of new classes of nanoparticle-enhanced materials, such as nanofluids and NPCM, has increased interest in understanding the impact of nanoparticles on macroscopic properties of the materials.

## References

[B1] Abdelhamid M. Attia Y. A. Abdel-Harith M. (2020). The significance of nano-shapes in nanoparticle-enhanced laser-induced breakdown spectroscopy. J. Anal. A. T. Spectrom. 35, 2982–2989. 10.1039/d0ja00329h

[B2] Aiyyzhy K. O. Barmina E. V. Rakov I. I. Voronov V. V. Shafeev G. A. (2023). Laser synthesis of ruby and its nanoparticles for photo-conversion of solar spectrum. Laser Phys. Lett. 20, 046001. 10.1088/1612-202x/acb708

[B3] Aiyyzhy K. O. Barmina E. V. Voronov V. V. Shafeev G. A. Novikov G. G. Uvarov O. V. (2022a). Laser ablation and fragmentation of Boron in liquids. Opt. Laser Technol. 155, 108393. 10.1016/j.optlastec.2022.108393

[B4] Aiyyzhy K. O. Barmina E. V. Voronov V. V. Shafeev G. A. Novikov G. G. Uvarov O. V. (2022b). Laser ablation and fragmentation of Boron in liquids. Opt. Laser Technol. 155, 108393. 10.1016/j.optlastec.2022.108393

[B5] Akman E. Aktas O. C. Genc Oztoprak B. Gunes M. Kacar E. Gundogdu O. (2013). Fragmentation of the gold nanoparticles using femtosecond Ti:Sapphire laser and their structural evolution. Opt. Laser Technol. 49, 156–160. 10.1016/j.optlastec.2013.01.003

[B6] Alavi S. Thompson D. L. (2006). Molecular dynamics simulations of the melting of aluminum nanoparticles. J. Phys. Chem. A 110, 1518–1523. 10.1021/jp053318s 16435812

[B7] AlMalki F. A. Khashan K. S. Jabir M. S. Hadi A. A. Sulaiman G. M. Abdulameer F. A. (2022). Eco‐friendly synthesis of carbon nanoparticles by laser ablation in water and evaluation of their antibacterial activity. J. Nanomater 2022, 7927447. 10.1155/2022/7927447

[B8] Alsaba M. T. Al Dushaishi M. F. Abbas A. K. (2020). A comprehensive review of nanoparticles applications in the oil and gas industry. J. Pet. Explor Prod. Technol. 10, 1389–1399. 10.1007/s13202-019-00825-z

[B9] Amendola V. Scaramuzza S. Carraro F. Cattaruzza E. (2017). Formation of alloy nanoparticles by laser ablation of Au/Fe multilayer films in liquid environment. J. Colloid Interface Sci. 489, 18–27. 10.1016/j.jcis.2016.10.023 27770998

[B10] Arefev M. I. Shugaev M. V. Zhigilei L. V. (2022). Kinetics of laser-induced melting of thin gold film: how slow can it get? Sci. Adv. 8, eabo2621–15. 10.1126/sciadv.abo2621 36129986 PMC9491712

[B11] Ashikkalieva K. K. Kononenko V. V. Arutyunyan N. R. Akhlyustina E. V. Zavedeev E. V. Vasiliev A. L. (2023). Laser synthesis of gold nanochains from hydrochloroauric acid aqueous solutions. Phys. Wave Phenom. 31, 44–50. 10.3103/s1541308x23010016

[B12] Ashikkalieva K. K. Kononenko V. V. Vasil’ev A. L. Akhlyustina E. V. Gololobov V. M. Arutyunyan N. R. (2022). Synthesis of gold nanoparticles from aqueous solutions of hydrochloroauric acid under multipulse femtosecond irradiation. Phys. Wave Phenom. 30, 17–24. 10.3103/s1541308x22010046

[B13] Astafyeva L. G. Pustovalov V. K. Fritzsche W. (2017). Characterization of plasmonic and thermo-optical parameters of spherical metallic nanoparticles. Nano-Structures Nano-Objects 12, 57–67. 10.1016/j.nanoso.2017.08.014

[B14] Baimler I. V. Simakin A. V. Dikovskaya A. O. Voronov V. V. Uvarov O. V. Smirnov A. A. (2024). Fabrication and growth mechanism of t-selenium nanorods during laser ablation and fragmentation in organic liquids. Front. Chem. 12, 1449570. 10.3389/fchem.2024.1449570 39371596 PMC11449723

[B15] Barcikowski S. Menéndez-Manjón A. Chichkov B. Brikas M. Račiukaitis G. (2007). Generation of nanoparticle colloids by picosecond and femtosecond laser ablations in liquid flow. Appl. Phys. Lett. 91. 10.1063/1.2773937

[B16] Barmina E. V. Shafeev G. A. Kuzmin P. G. Serkov A. A. Simakin A. V. Melnik N. N. (2014). Laser-assisted generation of gold nanoparticles and nanostructures in liquid and their plasmonic luminescence. Appl. Phys. A 115, 747–752. 10.1007/s00339-014-8385-4

[B17] Bhardwaj B. Singh P. Kumar A. Kumar S. Budhwar V. (2020). Eco-friendly greener synthesis of nanoparticles. Adv. Pharm. Bull. 10, 566–576. 10.34172/apb.2020.067 33072534 PMC7539319

[B18] Bongiovanni G. Olshin P. K. Yan C. Voss J. M. Drabbels M. Lorenz U. J. (2021). The fragmentation mechanism of gold nanoparticles in water under femtosecond laser irradiation. Nanoscale Adv. 3, 5277–5283. 10.1039/d1na00406a 34589666 PMC8439145

[B19] Buongiorno J. Hu L.-W. Kim S. J. Hannink R. Truong B. A. O. Forrest E. (2008). Nanofluids for enhanced economics and safety of nuclear reactors: an evaluation of the potential features, issues, and research gaps. Nucl. Technol. 162, 80–91. 10.13182/nt08-a3934

[B20] Catone D. Ciavardini A. Di Mario L. Paladini A. Toschi F. Cartoni A. (2018). Plasmon controlled shaping of metal nanoparticle aggregates by femtosecond laser-induced melting. J. Phys. Chem. Lett. 9, 5002–5008. 10.1021/acs.jpclett.8b02117 30107131

[B21] Chen X. Gao H. Tang Z. Dong W. Li A. Wang G. (2020). Optimization strategies of composite phase change materials for thermal energy storage, transfer, conversion and utilization. Energy Environ. Sci. 13, 4498–4535. 10.1039/d0ee01355b

[B22] Chen X. Munjiza A. Zhang K. Wen D. (2014). Molecular dynamics simulation of heat transfer from a gold nanoparticle to a water pool. J. Phys. Chem. C 118, 1285–1293. 10.1021/jp410054j

[B23] Chen X. Zhang M. Zhu J. Tao Z. Qiu L. (2023). Laser sintering of Cu nanoparticles deposited on ceramic substrates: experiments and modeling. Addit. Manuf. 69, 103527. 10.1016/j.addma.2023.103527

[B24] Colla L. Fedele L. Mancin S. Danza L. Manca O. (2017). Nano-PCMs for enhanced energy storage and passive cooling applications. Appl. Therm. Eng. 110, 584–589. 10.1016/j.applthermaleng.2016.03.161

[B25] Cui J. Yang L. Wang Y. (2013). Molecular dynamics simulation study of the melting of silver nanoparticles. Integr. Ferroelectr. 145, 1–9. 10.1080/10584587.2013.787873

[B26] Das S. K. Choi S. U. S. Patel H. E. (2006). Heat transfer in nanofluids—a review. Heat. Transf. Eng. 27, 3–19. 10.1080/01457630600904593

[B27] Day E. S. Morton J. G. West J. L. (2009). Nanoparticles for thermal cancer therapy. J. Biomech. Eng. 131, 074001. 10.1115/1.3156800 19640133

[B28] De Giacomo A. De Bonis A. Dell’Aglio M. De Pascale O. Gaudiuso R. Orlando S. (2011). Laser ablation of graphite in water in a range of pressure from 1 to 146 atm using single and double pulse techniques for the production of carbon nanostructures. J. Phys. Chem. C 115, 5123–5130. 10.1021/jp109389c

[B29] Dolgaev S. I. Simakin A. V. Voronov V. V. Shafeev G. A. Bozon-Verduraz F. (2002). Nanoparticles produced by laser ablation of solids in liquid environment. Appl. Surf. Sci. 186, 546–551. 10.1016/s0169-4332(01)00634-1

[B30] Duan H. Xue Y. Cui Z. Fu Q. Chen X. Zhang R. (2018). Influence of size on melting thermodynamics of nanoparticles: mechanism, factors, range, and degree. Part Part Syst. Charact. 35, 1800156. 10.1002/ppsc.201800156

[B31] Dzienny P. Szczęsny R. Rerek T. Trzciński M. Skowroński A. A. Antończak A. (2022). Laser-induced alloy nanoparticles on Au-Sn thin layers. Appl. Surf. Sci. 591, 153147. 10.1016/j.apsusc.2022.153147

[B32] Ekici O. Harrison R. K. Durr N. J. Eversole D. S. Lee M. Ben-Yakar A. (2008). Thermal analysis of gold nanorods heated with femtosecond laser pulses. J. Phys. D. Appl. Phys. 41, 185501. 10.1088/0022-3727/41/18/185501 21799542 PMC3143716

[B33] Erutin D. Popovich A. Sufiiarov V. (2023). Selective laser melting of (Fe-Si-B)/Cu composite: structure and magnetic properties study. Met. (Basel) 13, 428. 10.3390/met13020428

[B34] Fakhrutdinova E. D. Volokitina A. V. Kulinich S. A. Goncharova D. A. Kharlamova T. S. Svetlichnyi V. A. (2024). Plasmonic nanocomposites of ZnO-Ag produced by laser ablation and their photocatalytic destruction of rhodamine, tetracycline and phenol. Mater. (Basel) 17, 527. 10.3390/ma17020527 PMC10818360 38276466

[B35] Fazio E. Saija R. Santoro M. Abir S. Neri F. Tommasini M. (2020). On the optical properties of Ag–Au colloidal alloys pulsed laser ablated in liquid: experiments and theory. J. Phys. Chem. C 124, 24930–24939. 10.1021/acs.jpcc.0c05270

[B36] Golubovskaya A. G. Goncharova D. A. Fakhrutdinova E. D. Kharlamova T. S. Vodyankina O. V. Svetlichnyi V. A. (2024). Photocatalytic activity of colloidal Bi–Si-based nanoparticles prepared by laser synthesis in liquid. Mater Chem. Phys. 314, 128800. 10.1016/j.matchemphys.2023.128800

[B37] Gorin D. A. Portnov S. A. Inozemtseva O. A. Luklinska Z. Yashchenok A. M. Pavlov A. M. (2008). Magnetic/gold nanoparticle functionalized biocompatible microcapsules with sensitivity to laser irradiation. Phys. Chem. Chem. Phys. 10, 6899–6905. 10.1039/b809696a 19015796

[B38] Gudkov S. V. Sarimov R. M. Astashev M. E. Pishchalnikov R. Y. Yanykin D. V. Simakin A. V. (2024). Modern physical methods and technologies in agriculture. Physics–Uspekhi 67, 194–210. 10.3367/UFNe.2023.09.039577

[B39] Guillén G. G. Palma M. I. M. Krishnan B. Avellaneda D. Castillo G. A. Roy T. D. (2015). Structure and morphologies of ZnO nanoparticles synthesized by pulsed laser ablation in liquid: effects of temperature and energy fluence. Mater Chem. Phys. 162, 561–570. 10.1016/j.matchemphys.2015.06.030

[B40] Hadžić B. Vasić B. Matović B. Kuryliszyn-Kudelska I. Dobrowolski W. Romčević M. (2018). Influence of laser-induced heating on MnO nanoparticles. J. Raman Spectrosc. 49, 817–821. 10.1002/jrs.5358

[B41] Hashemi F. Hormozi-Nezhad M. R. Corbo C. Farvadi F. Shokrgozar M. A. Mehrjoo M. (2019). Laser irradiation affects the biological identity and cellular uptake of plasmonic nanoparticles. Nanoscale 11, 5974–5981. 10.1039/c8nr09622h 30892307

[B42] Heilweil E. J. Hochstrasser R. M. (1985). Nonlinear spectroscopy and picosecond transient grating study of colloidal gold. J. Chem. Phys. 82, 4762–4770. 10.1063/1.448693

[B43] Hidayah A. N. Herbani Y. (2020). Femtosecond laser melting and alloying in Au-Ag nanoalloys from colloid mixture of gold-silver nanoparticles. AIP Conf. Proc. 2256, 020006. 10.1063/5.0018421

[B44] Hodak J. H. Martini I. Hartland G. V. (1998). Spectroscopy and dynamics of nanometer-sized noble metal particles. J. Phys. Chem. B 102, 6958–6967. 10.1021/jp9809787

[B45] Hodges J. M. Morse J. R. Fenton J. L. Ackerman J. D. Alameda L. T. Schaak R. E. (2017). Insights into the seeded-growth synthesis of colloidal hybrid nanoparticles. Chem. Mater 29, 106–119. 10.1021/acs.chemmater.6b02795

[B46] Honda M. Saito Y. Smith N. I. Fujita K. Kawata S. (2011). Nanoscale heating of laser irradiated single gold nanoparticles in liquid. Opt. Express 19, 12375. 10.1364/oe.19.012375 21716475

[B47] Huang H. Sivayoganathan M. Duley W. W. Zhou Y. (2015). Efficient localized heating of silver nanoparticles by low-fluence femtosecond laser pulses. Appl. Surf. Sci. 331, 392–398. 10.1016/j.apsusc.2015.01.086

[B48] Inasawa S. Sugiyama M. Yamaguchi Y. (2005). Laser-induced shape transformation of gold nanoparticles below the melting point: the effect of surface melting. J. Phys. Chem. B 109, 3104–3111. 10.1021/jp045167j 16851329

[B49] Iqbal A. Mahmoud M. S. Sayed E. T. Elsaid K. Abdelkareem M. A. Alawadhi H. (2021). Evaluation of the nanofluid-assisted desalination through solar stills in the last decade. J. Environ. Manage 277, 111415. 10.1016/j.jenvman.2020.111415 33010657

[B50] Izadi M. Assad M. E. H. (2024). “Use of nanofluids in solar energy systems,” in Design and Performance optimization of renewable energy systems (Germany: Elsevier), 221–250.

[B51] Jiang R. Yang M. Meng J. Zhao P. Liu P. Zheng X. (2023). Enhanced catalytic performance of CuNi bimetallic nanoparticles for hydrogen evolution from ammonia borane hydrolysis. Int. J. Hydrogen Energy 48, 18245–18256. 10.1016/j.ijhydene.2023.01.316

[B52] Jiang Z. Li L. Huang H. He W. Ming W. (2022). Progress in laser ablation and biological synthesis processes: “top-down” and “bottom-up” approaches for the green synthesis of Au/Ag nanoparticles. Int. J. Mol. Sci. 23, 14658. 10.3390/ijms232314658 36498986 PMC9736509

[B53] Kaningini A. G. Nelwamondo A. M. Azizi S. Maaza M. Mohale K. C. (2022). Metal nanoparticles in agriculture: a review of possible use. Coatings 12, 1586. 10.3390/coatings12101586

[B54] Kefeni K. K. Mamba B. B. Msagati T. A. M. (2017). Application of spinel ferrite nanoparticles in water and wastewater treatment: a review. Sep. Purif. Technol. 188, 399–422. 10.1016/j.seppur.2017.07.015

[B55] Khan M. Al-Hamoud K. Liaqat Z. Shaik M. R. Adil S. F. Kuniyil M. (2020). Synthesis of au, ag, and au–ag bimetallic nanoparticles using pulicaria undulata extract and their catalytic activity for the reduction of 4-nitrophenol. Nanomaterials 10, 1885. 10.3390/nano10091885 32962292 PMC7559643

[B56] Khodadadi J. M. Fan L. Babaei H. (2013). Thermal conductivity enhancement of nanostructure-based colloidal suspensions utilized as phase change materials for thermal energy storage: a review. Renew. Sustain Energy Rev. 24, 418–444. 10.1016/j.rser.2013.03.031

[B57] Kirichenko N. A. Sukhov I. A. Shafeev G. A. Shcherbina M. E. (2012). Evolution of the distribution function of Au nanoparticles in a liquid under the action of laser radiation. Quantum Electron 42, 175–180. 10.1070/qe2012v042n02abeh014779

[B58] Kojima M. Koshizaki N. Ishikawa Y. (2020). Laser-induced nano-heater performance of B4C submicrometer spherical particles fabricated by pulsed laser melting in liquid. Appl. Nanosci. 10, 1853–1860. 10.1007/s13204-020-01276-3

[B59] Kovalev M. Nastulyavichus A. Podlesnykh I. Stsepuro N. Pryakhina V. Greshnyakov E. (2023). Au-hyperdoped Si nanolayer: laser processing techniques and corresponding material properties. Mater. (Basel) 16, 4439. 10.3390/ma16124439 PMC10300740 37374622

[B60] Kozyukhin S. Lazarenko P. Vorobyov Y. Baranchikov A. Glukhenkaya V. Smayev M. (2019). Laser-induced modification and formation of periodic surface structures (ripples) of amorphous GST225 phase change materials. Opt. Laser Technol. 113, 87–94. 10.1016/j.optlastec.2018.12.017

[B61] Kranz D. Bessel P. Niemeyer M. Borg H. Rosebrock M. Himstedt R. (2022). Size-dependent threshold of the laser-induced phase transition of colloidally dispersed copper oxide nanoparticles. J. Phys. Chem. C 126, 15263–15273. 10.1021/acs.jpcc.2c03815

[B62] Křenek T. Vála L. Medlín R. Pola J. Jandová V. Vavruňková V. (2022). A novel route of colloidal chemistry: room temperature reactive interactions between titanium monoxide and silicon monoxide sols produced by laser ablation in liquid resulting in the formation of titanium disilicide. Dalt Trans. 51, 13831–13847. 10.1039/d2dt02065c 36039852

[B63] Krishna J. Kishore P. S. Solomon A. B. (2017). Heat pipe with nano enhanced-PCM for electronic cooling application. Exp. Therm. Fluid Sci. 81, 84–92. 10.1016/j.expthermflusci.2016.10.014

[B64] Kucherik A. Samyshkin V. Prusov E. Osipov A. Panfilov A. Buharov D. (2021). Formation of fractal dendrites by laser-induced melting of aluminum alloys. Nanomaterials 11, 1043–1048. 10.3390/nano11041043 33921684 PMC8074200

[B65] Kumar N. Sonawane S. S. Sonawane S. H. (2018). Experimental study of thermal conductivity, heat transfer and friction factor of Al2O3 based nanofluid. Int. Commun. Heat. Mass Transf. 90, 1–10. 10.1016/j.icheatmasstransfer.2017.10.001

[B66] Kuzmin P. G. Shafeev G. A. Voronov V. V. Raspopov R. V. Arianova E. A. Trushina E. N. (2012). Bioavailable nanoparticles obtained in laser ablation of a selenium target in water. Quantum Electron 42, 1042–1044. 10.1070/qe2012v042n11abeh014754

[B67] Letfullin R. R. George T. F. Duree G. C. Bollinger B. M. (2008). Ultrashort laser pulse heating of nanoparticles: comparison of theoretical approaches. Adv. Opt. Technol. 2008. 10.1155/2008/251718

[B68] Lim J. Kim Y. Shin J. Lee Y. Shin W. Qu W. (2020). Continuous-wave laser-induced transfer of metal nanoparticles to arbitrary polymer substrates. Nanomaterials 10, 701–710. 10.3390/nano10040701 32272614 PMC7221800

[B69] Lin S. C. Al-Kayiem H. H. (2016). Evaluation of copper nanoparticles–Paraffin wax compositions for solar thermal energy storage. Sol. Energy 132, 267–278. 10.1016/j.solener.2016.03.004

[B70] Link S. Burda C. Nikoobakht B. El-Sayed M. A. (2000a). Laser-induced shape changes of colloidal gold nanorods using femtosecond and nanosecond laser pulses. J. Phys. Chem. B 104, 6152–6163. 10.1021/jp000679t

[B71] Link S. Burda C. Nikoobakht B. El-Sayed M. A. (2000b). Laser-induced shape changes of colloidal gold nanorods using femtosecond and nanosecond laser pulses. J. Phys. Chem. B 104, 6152–6163. 10.1021/jp000679t

[B72] Linz N. Freidank S. Liang X.-X. Vogel A. (2016). Wavelength dependence of femtosecond laser-induced breakdown in water and implications for laser surgery. Phys. Rev. B 94, 024113. 10.1103/physrevb.94.024113

[B73] Liu B. Wildman R. Tuck C. Ashcroft I. Hague R. (2011). Investigation the effect of particle size distribution on processing parameters optimisation in selective laser melting process. 22nd Annu. Int Solid Free Fabr Symp - An Addit Manuf Conf SFF 2011, 227–238. 10.26153/tsw/15290

[B74] Liu G. Liu D. (2019). Noncontact direct temperature and concentration profiles measurement of soot and metal-oxide nanoparticles in optically thin/thick nanofluid fuel flames. Int. J. Heat. Mass Transf. 134, 237–249. 10.1016/j.ijheatmasstransfer.2019.01.035

[B75] Llamosa P. D. Espinosa A. Martínez L. Roman E. Ballesteros C. Mayoral A. (2013). Thermal diffusion at nanoscale: from CoAu alloy nanoparticles to Co@ Au core/shell structures. J. Phys. Chem. C 117, 3101–3108. 10.1021/jp310971f

[B76] Lombard J. Biben T. Merabia S. (2014). Kinetics of nanobubble generation around overheated nanoparticles. Phys. Rev. Lett. 112, 105701. 10.1103/physrevlett.112.105701 24679307

[B77] Lu J. Zhuo L. (2023). Additive manufacturing of titanium alloys via selective laser melting: fabrication, microstructure, post-processing, performance and prospect. Int. J. Refract Met. Hard Mater 111, 106110. 10.1016/j.ijrmhm.2023.106110

[B78] Lu Y. Zhou Y. Wen P. Luo F. Cao J. Xu Y. (2023). Effect of laser power on microstructure and mechanical properties of K418 nickel-based alloy prepared by selective laser melting. J. Mater Res. Technol. 27, 2964–2975. 10.1016/j.jmrt.2023.10.189

[B79] Ma C. Chen L. Cao C. Li X. (2017). Nanoparticle-induced unusual melting and solidification behaviours of metals. Nat. Commun. 8, 14178–14187. 10.1038/ncomms14178 28098147 PMC5253640

[B80] Ma M. Zhang X. Qing S. Wang H. (2024a). Wettability-dependent thermal transport at the Fe nanoparticle-water interface: molecular dynamics simulations. J. Mol. Liq. 402, 124717. 10.1016/j.molliq.2024.124717

[B81] Ma Y. Li K. Li C. Miao X. Araki T. Wu M. (2024b). Corrosion behavior of selective laser melted 6061 aluminum alloy electrodes for aluminum-air battery. J. Power Sources 594, 233999. 10.1016/j.jpowsour.2023.233999

[B82] Mafuné F. Kohno J. Y. Takeda Y. Kondow T. (2001). Dissociation and aggregation of gold nanoparticles under laser irradiation. J. Phys. Chem. B 105, 9050–9056. 10.1021/jp0111620

[B83] Mafuné F. Kohno J. Y. Takeda Y. Kondow T. (2003). Formation of gold nanonetworks and small gold nanoparticles by irradiation of intense pulsed laser onto gold nanoparticles. J. Phys. Chem. B 107, 12589–12596. 10.1021/jp030173l

[B84] Maximova K. Aristov A. Sentis M. Kabashin A. V. (2015). Size-controllable synthesis of bare gold nanoparticles by femtosecond laser fragmentation in water. Nanotechnology 26, 065601. 10.1088/0957-4484/26/6/065601 25605000

[B85] Mayelifartash A. Abdol M. A. Sadeghzadeh S. (2021). Thermal conductivity and interfacial thermal resistance behavior for the polyaniline–boron carbide heterostructure. Phys. Chem. Chem. Phys. 23, 13310–13322. 10.1039/d1cp00562f 34095909

[B86] Menéndez-Manjón A. Chichkov B. N. Barcikowski S. (2010). Influence of water temperature on the hydrodynamic diameter of gold nanoparticles from laser ablation. J. Phys. Chem. C 114, 2499–2504. 10.1021/jp909897v

[B87] Metwally K. Mensah S. Baffou G. (2015). Fluence threshold for photothermal bubble generation using plasmonic nanoparticles. J. Phys. Chem. C 119, 28586–28596. 10.1021/acs.jpcc.5b09903

[B88] Mikami K. Aizuka M. Setogawa H. Saito N. Murakmi Y. (2021). Preparation of 9, 10-Bis (Phenylethynyl) anthracene and 1-Chloro-9, 10-Bis (Phenylethynyl) anthracene nanoparticles using the laser processing in liquids: influence of the surfactants on the optical properties. J. Mol. Struct. 1246, 131215. 10.1016/j.molstruc.2021.131215

[B89] Mintcheva N. Yamaguchi S. Kulinich S. A. (2020). Hybrid TiO2-ZnO nanomaterials prepared using laser ablation in liquid. Mater. (Basel) 13, 719. 10.3390/ma13030719 PMC7040934 32033417

[B90] Moita A. Moreira A. Pereira J. (2021). Nanofluids for the next generation thermal management of electronics: a review. Symmetry (Basel) 13, 1362. 10.3390/sym13081362

[B91] Moore J. A. Chow J. C. L. (2021). Recent progress and applications of gold nanotechnology in medical biophysics using artificial intelligence and mathematical modeling. Nano Express 2, 022001. 10.1088/2632-959x/abddd3

[B92] Muñeton Arboleda D. Santillán J. M. J. Mendoza Herrera L. J. Van Raap M. B. F. Mendoza Zélis P. Muraca D. (2015). Synthesis of Ni nanoparticles by femtosecond laser ablation in liquids: structure and sizing. J. Phys. Chem. C 119, 13184–13193. 10.1021/acs.jpcc.5b03124

[B93] Munyalo J. M. Zhang X. (2018). Particle size effect on thermophysical properties of nanofluid and nanofluid based phase change materials: a review. J. Mol. Liq. 265, 77–87. 10.1016/j.molliq.2018.05.129

[B94] Nag A. Nguyen C. M. Tibbetts K. M. (2023). Heterogeneous to homogeneous Cu–Ag nanoparticles by laser reduction in liquid. Appl. Surf. Sci. 610, 155384. 10.1016/j.apsusc.2022.155384

[B95] Nanda K. K. (2009). Size-dependent melting of nanoparticles: hundred years of thermodynamic model. Pramana 72, 617–628. 10.1007/s12043-009-0055-2

[B96] Nedyalkov N. N. Imamova S. E. Atanasov P. A. Toshkova R. A. Gardeva E. G. Yossifova L. S. (2011). Interaction of gold nanoparticles with nanosecond laser pulses: nanoparticle heating. Appl. Surf. Sci. 257, 5456–5459. 10.1016/j.apsusc.2010.11.010

[B97] Niozu A. Kumagai Y. Fukuzawa H. Yokono N. You D. Saito S. (2021). Relation between inner structural dynamics and ion dynamics of laser-heated nanoparticles. Phys. Rev. X (11), 031046–31112. 10.1103/PhysRevX.11.031046

[B98] Noack J. Vogel A. (1999). Laser-induced plasma formation in water at nanosecond to femtosecond time scales: calculation of thresholds, absorption coefficients, and energy density. IEEE J. Quantum Electron 35, 1156–1167. 10.1109/3.777215

[B99] Okamoto T. Nakamura T. Sakota K. Yatsuhashi T. (2019). Synthesis of single-nanometer-sized gold nanoparticles in liquid–liquid dispersion system by femtosecond laser irradiation. Langmuir 35, 12123–12129. 10.1021/acs.langmuir.9b01854 31446759

[B100] Omelchenko A. I. Sobol E. N. Simakin A. V. Serkov A. A. Sukhov I. A. Shafeev G. A. (2015). Biofunctional magnetic ‘core–shell’nanoparticles generated by laser ablation of iron in liquid. Laser Phys. 25, 025607. 10.1088/1054-660x/25/2/025607

[B101] Paskhin M. O. Aiyyzhy K. O. Pobedonostsev R. V. Kazantseva D. V. Rakov I. I. Barmina E. V. (2023). Ruby nanoparticles for greenhouse farming: synthesis, features and application. J. Compos Sci. 8, 7. 10.3390/jcs8010007

[B102] Pavlov I. S. Barmina E. V. Zhilnikova M. I. Shafeev G. A. Zinin P. V. Filonenko V. P. (2022). Production of spherical boron-carbide particles encapsulated in a graphite shell. Nanobiotechnology Rep. 17, 290–296. 10.1134/s2635167622030132

[B103] Pelton M. Sader J. E. Burgin J. Liu M. Guyot-Sionnest P. Gosztola D. (2009). Damping of acoustic vibrations in gold nanoparticles. Nat. Nanotechnol. 4, 492–495. 10.1038/nnano.2009.192 19662009

[B104] Pembere A. M. S. Wu H. An P. Magero D. Louis H. Luo Z. (2022). Guerbet coupling of methanol catalysed by titanium clusters. Chem. Phys. Lett. 802, 139719. 10.1016/j.cplett.2022.139719

[B105] Plech A. Kotaidis V. Grésillon S. Dahmen C. Von Plessen G. (2004). Laser-induced heating and melting of gold nanoparticles studied by time-resolved x-ray scattering. Phys. Rev. B - Condens Matter Mater Phys. 70, 195423–195427. 10.1103/PhysRevB.70.195423

[B106] Plech A. Tack M. Huang H. Arefev M. Ziefuss A. R. Levantino M. (2023). Physical regimes and mechanisms of picosecond laser fragmentation of gold nanoparticles in water from X-ray probing and atomistic simulations. ACS Nano 18, 10527–10541. 10.1021/acsnano.3c12314 38567906

[B107] Plech A. Ziefuß A. R. Levantino M. Streubel R. Reich S. Reichenberger S. (2022). Low efficiency of laser heating of gold particles at the plasmon resonance: an x-ray calorimetry study. ACS Photonics 9, 2981–2990. 10.1021/acsphotonics.2c00588

[B108] Poborchii V. V. Kolobov A. V. Tanaka K. (1999). Photomelting of selenium at low temperature. Appl. Phys. Lett. 74, 215–217. 10.1063/1.123297

[B109] Pustovalov V. K. (2006). Investigation of threshold laser intensities for melting and evaporation of spherical and spheroidal nanoparticles in media by short laser pulses. Chem. Phys. Lett. 421, 142–147. 10.1016/j.cplett.2006.01.063

[B110] Pustovalov V. K. (2024). Heating of nanoparticles and their environment by laser radiation and applications. Nanotechnol. Precis. Eng. 7. 10.1063/10.0022560

[B111] Pustovalov V. K. Smetannikov A. S. Zharov V. P. (2008). Photothermal and accompanied phenomena of selective nanophotothermolysis with gold nanoparticles and laser pulses. Laser Phys. Lett. 5, 775–792. 10.1002/lapl.200810072

[B112] Pyatenko A. Wang H. Koshizaki N. Tsuji T. (2013). Mechanism of pulse laser interaction with colloidal nanoparticles. Laser Phot. Rev. 7, 596–604. 10.1002/lpor.201300013

[B113] Qin Z. Bischof J. C. (2012). Thermophysical and biological responses of gold nanoparticle laser heating. Chem. Soc. Rev. 41, 1191–1217. 10.1039/c1cs15184c 21947414

[B114] Rafati M. Hamidi A. A. Niaser M. S. (2012). Application of nanofluids in computer cooling systems (heat transfer performance of nanofluids). Appl. Therm. Eng. 45, 9–14.

[B115] Ramesh G. Prabhu N. K. (2011). Review of thermo-physical properties, wetting and heat transfer characteristics of nanofluids and their applicability in industrial quench heat treatment. Nanoscale Res. Lett. 6, 334–415. 10.1186/1556-276x-6-334 21711877 PMC3211422

[B116] Rico C. M. Peralta-Videa J. R. Gardea-Torresdey J. L. (2015). Chemistry, biochemistry of nanoparticles, and their role in antioxidant defense system in plants. Nanotechnol. plant Sci. nanoparticles their impact plants, 1–17. 10.1007/978-3-319-14502-0_1

[B117] Riley R. S. Day E. S. (2017). Gold nanoparticle‐mediated photothermal therapy: applications and opportunities for multimodal cancer treatment. Wiley Interdiscip. Rev. Nanomedicine Nanobiotechnology 9, e1449. 10.1002/wnan.1449 PMC5474189 28160445

[B118] Saha K. Agasti S. S. Kim C. Li X. Rotello V. M. (2012). Gold nanoparticles in chemical and biological sensing. Chem. Rev. 112, 2739–2779. 10.1021/cr2001178 22295941 PMC4102386

[B119] Sajjadi M. A. Modabberifar M. Taheri M. Badrossamay M. Hemmati M. (2024). An experimental investigation of selective laser melting parameters effects on ferromagnetic properties of pure iron in both the as-built and annealed conditions. J. Magn. Magn. Mater 596, 171924. 10.1016/j.jmmm.2024.171924

[B120] Sakaguchi Y. Tamura K. (2021). Photo-induced effects on amorphous and liquid selenium by pulsed laser illumination: photo-induced structural changes in a network of selenium chains. Z. für Phys. Chem. 235, 189–212. 10.1515/zpch-2020-1650

[B121] Saraeva I. N. Kudryashov S. I. Rudenko A. A. Zhilnikova M. I. Ivanov D. S. Zayarny D. A. (2019). Effect of fs/ps laser pulsewidth on ablation of metals and silicon in air and liquids, and on their nanoparticle yields. Appl. Surf. Sci. 470, 1018–1034. 10.1016/j.apsusc.2018.11.199

[B122] Schmidl G. Raugust M. Jia G. Dellith A. Dellith J. Schmidl F. (2022). Porous spherical gold nanoparticles via a laser induced process. Nanoscale Adv. 4, 4122–4130. 10.1039/d2na00396a 36285216 PMC9514562

[B123] Sdvizhenskii P. A. Lednev V. N. (2022). Combined nano-and microsecond laser ablation for elemental depth profiling of metal targets by laser-induced breakdown spectroscopy. Phys. Wave Phenom. 30, 37–43. 10.3103/s1541308x22010095

[B124] Serkov A. A. Barmina E. V. Kuzmin P. G. Shafeev G. A. (2015b). Self-assembly of nanoparticles into nanowires under laser exposure in liquids. Chem. Phys. Lett. 623, 93–97. 10.1016/j.cplett.2015.01.050

[B125] Serkov A. A. Barmina E. V. Shafeev G. A. Voronov V. V. (2015c). Laser ablation of titanium in liquid in external electric field. Appl. Surf. Sci. 348, 16–21. 10.1016/j.apsusc.2014.12.139

[B126] Serkov A. A. Shcherbina M. E. Kuzmin P. G. Kirichenko N. A. (2015a). Laser-induced agglomeration of gold nanoparticles dispersed in a liquid. Appl. Surf. Sci. 336, 96–102. 10.1016/j.apsusc.2014.09.173

[B127] Sharif A. Farid N. O’Connor G. M. (2022). Ultrashort laser sintering of metal nanoparticles: a review. Results Eng. 16, 100731. 10.1016/j.rineng.2022.100731

[B128] Shibuta Y. Suzuki T. (2007). Melting and nucleation of iron nanoparticles: a molecular dynamics study. Chem. Phys. Lett. 445, 265–270. 10.1016/j.cplett.2007.07.098

[B129] Simakin A. V. Astashev M. E. Baimler I. V. Uvarov O. V. Voronov V. V. Vedunova M. V. (2019). The effect of gold nanoparticle concentration and laser fluence on the laser-induced water decomposition. J. Phys. Chem. B 123, 1869–1880. 10.1021/acs.jpcb.8b11087 30696249

[B130] Simakin A. V. Baimler I. V. Smirnova V. V. Uvarov O. V. Kozlov V. A. Gudkov S. V. (2021). Evolution of the size distribution of gold nanoparticles under laser irradiation. Phys. Wave Phenom. 29, 102–107. 10.3103/S1541308X21020126

[B131] Singh S. C. Mishra S. K. Srivastava R. K. Gopal R. (2010). Optical properties of selenium quantum dots produced with laser irradiation of water suspended Se nanoparticles. J. Phys. Chem. C 114, 17374–17384. 10.1021/jp105037w

[B132] Sobhan M. A. Ams M. Withford M. J. Goldys E. M. (2010). Ultrafast laser ablative generation of gold nanoparticles: the influence of pulse energy, repetition frequency and spot size. J. Nanoparticle Res. 12, 2831–2842. 10.1007/s11051-010-9868-7

[B133] Sokolovskaya O. I. Sergeeva E. A. Golovan L. A. Kashkarov P. K. Khilov A. V. Kurakina D. A. (2021). Numerical simulation of enhancement of superficial tumor laser hyperthermia with silicon nanoparticles. Photonics (MDPI) 8, 580. 10.3390/photonics8120580

[B134] Stone M. B. Kolesnikov A. I. Fanelli V. R. May A. F. Bai S. Liu J. (2024). Characterization of aluminum and boron carbide based additive manufactured material for thermal neutron shielding. Mater Des. 237, 112463. 10.1016/j.matdes.2023.112463

[B135] Swiatkowska-Warkocka Z. Pyatenko A. Koga K. Kawaguchi K. Wang H. Koshizaki N. (2017). Various morphologies/phases of gold-based nanocomposite particles produced by pulsed laser irradiation in liquid media: insight in physical processes involved in particles formation. J. Phys. Chem. C 121, 8177–8187. 10.1021/acs.jpcc.7b00187

[B136] Tabayashi Y. Sakaki S. Koshizaki N. Yamauchi Y. Ishikawa Y. (2021). Behavior of thermally induced nanobubbles during instantaneous particle heating by pulsed laser melting in liquid. Langmuir 37, 7167–7175. 10.1021/acs.langmuir.1c00736 34078084

[B137] Tack M. Usama M. Kazamer N. Exner K. S. Brodmann M. Barcikowski S. (2024). Continuous and scalable laser synthesis of atom clusters with tunable surface oxidation for electrocatalytic water splitting. ACS Appl. Energy Mater 7, 4057–4067. 10.1021/acsaem.4c00342

[B138] Tarasenko N. V. Butsen A. V. Nevar E. A. (2005). Laser-induced modification of metal nanoparticles formed by laser ablation technique in liquids. Appl. Surf. Sci. 247, 418–422. 10.1016/j.apsusc.2005.01.093

[B139] Tascini A. S. Armstrong J. Chiavazzo E. Fasano M. Asinari P. Bresme F. (2017). Thermal transport across nanoparticle–fluid interfaces: the interplay of interfacial curvature and nanoparticle–fluid interactions. Phys. Chem. Chem. Phys. 19, 3244–3253. 10.1039/c6cp06403e 28083587

[B140] Terentyuk G. S. Maslyakova G. N. Suleymanova L. V. Khlebtsov N. G. Khlebtsov B. N. Akchurin G. G. (2009). Laser-induced tissue hyperthermia mediated by gold nanoparticles: toward cancer phototherapy. J. Biomed. Opt. 14, 021016. 10.1117/1.3122371 19405729

[B141] Tsuji T. Yahata T. Yasutomo M. Igawa K. Tsuji M. Ishikawa Y. (2013). Preparation and investigation of the formation mechanism of submicron-sized spherical particles of gold using laser ablation and laser irradiation in liquids. Phys. Chem. Chem. Phys. 15, 3099–3107. 10.1039/c2cp44159d 23303286

[B142] Varlamova E. G. Plotnikov E. Y. Baimler I. V. Gudkov S. V. Turovsky E. A. (2023). Pilot study of cytoprotective mechanisms of selenium nanorods (SeNrs) under ischemia-like conditions on cortical astrocytes. Int. J. Mol. Sci. 24, 12217. 10.3390/ijms241512217 37569591 PMC10419292

[B143] Vasa P. Sharma R. Singh M. Dharmadhikari A. K. Dharmadhikari J. A. Mathur D. (2014). Generation of stable colloidal gold nanoparticles by ultrashort laser-induced melting and fragmentation. Mater Res. Express 1, 035028. 10.1088/2053-1591/1/3/035028

[B144] Wang J. J. Zheng R. T. Gao J. W. Chen G. (2012). Heat conduction mechanisms in nanofluids and suspensions. Nano Today 7, 124–136. 10.1016/j.nantod.2012.02.007

[B145] Wei S. Saitow K. (2012). *In situ* multipurpose time-resolved spectrometer for monitoring nanoparticle generation in a high-pressure fluid. Rev. Sci. Instrum. 83, 073110. 10.1063/1.4737886 22852674

[B146] Werner D. Hashimoto S. (2013). Controlling the pulsed-laser-induced size reduction of Au and Ag nanoparticles via changes in the external pressure, laser intensity, and excitation wavelength. Langmuir 29, 1295–1302. 10.1021/la3046143 23259708

[B147] Wilczewska A. Z. Niemirowicz K. Markiewicz K. H. Car H. (2012). Nanoparticles as drug delivery systems. Pharmacol. Rep. 64, 1020–1037. 10.1016/s1734-1140(12)70901-5 23238461

[B148] Xi L. Guo S. Ding K. Prashanth K. G. Sarac B. Eckert J. (2021). Effect of nanoparticles on morphology and size of primary silicon and property of selective laser melted Al-high Si content alloys. Vacuum 191, 110405. 10.1016/j.vacuum.2021.110405

[B149] Xiao M. Zheng S. Shen D. Duley W. W. Zhou Y. N. (2020). Laser-induced joining of nanoscale materials: processing, properties, and applications. Nano Today 35, 100959. 10.1016/j.nantod.2020.100959

[B150] Xing Y. Bai X.-H. Zhang Y. Hu G.-M. Gao L.-G. Qi P.-C. (2023). Facile synthesis of Ag-Co bimetallic nanoparticles decorated Fe3O4@ EDTA nanocomposites and their enhanced catalytic activity. J. Magn. Magn. Mater 579, 170857. 10.1016/j.jmmm.2023.170857

[B151] Yadavali S. Sandireddy V. P. Kalyanaraman R. (2016). Transformation of irregular shaped silver nanostructures into nanoparticles by under water pulsed laser melting. Nanotechnology 27, 195602–195618. 10.1088/0957-4484/27/19/195602 27041091

[B152] Yang W. Liang H. Ma S. Wang D. Huang J. (2019). Gold nanoparticle based photothermal therapy: development and application for effective cancer treatment. Sustain Mater Technol. 22, e00109. 10.1016/j.susmat.2019.e00109

[B153] Yang Z. Zhang C. Zhang H. Lu J. (2023). Transient electron temperature and density changes in water breakdown induced by femtosecond laser pulses. Opt. Commun. 546, 129803. 10.1016/j.optcom.2023.129803

[B154] Zhang W. Zhou Y. Ding Y. Song L. Yuan Q. Zhao W. (2022). Sintering mechanism of size-controllable Cu-Ag core–shell nanoparticles for flexible conductive film with high conductivity, antioxidation, and electrochemical migration resistance. Appl. Surf. Sci. 586, 152691. 10.1016/j.apsusc.2022.152691

[B155] Zhu D. Yan J. Xie J. Liang Z. Bai H. (2021). Ultrafast laser-induced atomic structure transformation of Au nanoparticles with improved surface activity. ACS Nano 15, 13140–13147. 10.1021/acsnano.1c02570 34313426

[B156] Zhu Y. Fu J. Zheng C. Ji Z. (2016). Effect of nanosecond pulse laser ablation on the surface morphology of Zr-based metallic glass. Opt. Laser Technol. 83, 21–27. 10.1016/j.optlastec.2016.03.021

[B157] Ziefuß A. R. Reichenberger S. Rehbock C. Chakraborty I. Gharib M. Parak W. J. (2018). Laser fragmentation of colloidal gold nanoparticles with high-intensity nanosecond pulses is driven by a single-step fragmentation mechanism with a defined educt particle-size threshold. J. Phys. Chem. C 122, 22125–22136. 10.1021/acs.jpcc.8b04374

